# Pentazocine dependence in a 57-year-old female patient

**DOI:** 10.1192/j.eurpsy.2025.967

**Published:** 2025-08-26

**Authors:** G. P. Leonte, R. M. Rusu-Andron, H. G. Coman, M. M. Manea

**Affiliations:** 1 Psihiatrie, Spitalul Clinic Județean de Urgență, Cluj Napoca, Romania

## Abstract

**Introduction:**

Pentazocine dependence, although rare, poses serious risks in patients with chronic pain, especially after surgical interventions. Misuse of prescribed opioid analgesics, such as Pentazocine, can lead to a wide range of medical and psychiatric complications. This case study explores the long-term effects of Pentazocine dependence in a patient with coexisting psychiatric conditions and chronic medical illnesses.

**Objectives:**

MAM, a 57-yer-old female patient, was first introduced to Pantozocine following pancreatic surgery in 1999 for Wirsung duct calcifications, a hereditary condition. Initially prescribed for pain relief, the patient increased her dose to 14-20 vials/day over six months. Psychiatric issues, including suicidal ideation and financial distress, emerged shortly after her addiction took hold.

**Methods:**

Between 2002 and 2024, the patient was admitted to psychiatric wards approximately 55 times. In 2003, after an accidental burn injury to her lower limbs, she began injecting Pentazocine directly into the wounds, as intramuscular administration no longer produced the desired effects. Her conditionworsened with multiple suicide attempts, including an overdose of 22 vials of Pentazocine, which she survived. During her hospitalizations, she presented with complications such as insulin dependent diabetes mellitus, mixed tissue disease, cervical spondylosis, and Raynaud’s syndrome. Multiple reconstructive surgeries were performed for the wounds caused by repeated injections.

**Results:**

Meanwhile, the patient followed methadone substitution treatment until 2008, later and until now she only requiring antidepressant treatment for recurrent depressive disorder, her current diagnosis.

**Conclusions:**

This case highlights the significant risks associated long-term opioid dependence, particularly in patients with medical conditions and psychiatric comorbities. Managing Pentazocine dependence requires careful monitoring of pain medication, early psychiatric intervention, and long-term follow-up. Comprehensive care, including psychological support and substitution therapies, is essential for improving prognosis in such cases.

**Disclosure of Interest:**

None Declared

